# Cuff-Less Blood Pressure Prediction from ECG and PPG Signals Using Fourier Transformation and Amplitude Randomization Preprocessing for Context Aggregation Network Training

**DOI:** 10.3390/bios12030159

**Published:** 2022-03-04

**Authors:** Treesukon Treebupachatsakul, Apivitch Boosamalee, Siratchakrit Shinnakerdchoke, Suejit Pechprasarn, Nuntachai Thongpance

**Affiliations:** 1 Department of Biomedical Engineering, School of Engineering, King Mongkut’s Institute of Technology Ladkrabang, Bangkok 10520, Thailand; treesukon.tr@kmitl.ac.th (T.T.); 61011260@kmitl.ac.th (A.B.); 61011333@kmitl.ac.th (S.S.); 2 College of Biomedical Engineering, Rangsit University, Pathum Thani 12000, Thailand; suejit.p@rsu.ac.th

**Keywords:** electrocardiogram, photoplethysmography, blood pressure measurement, context aggregation network, cuff-less blood pressure measurement

## Abstract

This research proposes an algorithm to preprocess photoplethysmography (PPG) and electrocardiogram (ECG) signals and apply the processed signals to the context aggregation network-based deep learning to achieve higher accuracy of continuous systolic and diastolic blood pressure monitoring than other reported algorithms. The preprocessing method consists of the following steps: (1) acquiring the PPG and ECG signals for a two second window at a sampling rate of 125 Hz; (2) separating the signals into an array of 250 data points corresponding to a 2 s data window; (3) randomizing the amplitude of the PPG and ECG signals by multiplying the 2 s frames by a random amplitude constant to ensure that the neural network can only learn from the frequency information accommodating the signal fluctuation due to instrument attachment and installation; (4) Fourier transforming the windowed PPG and ECG signals obtaining both amplitude and phase data; (5) normalizing both the amplitude and the phase of PPG and ECG signals using z-score normalization; and (6) training the neural network using four input channels (the amplitude and the phase of PPG and the amplitude and the phase of ECG), and arterial blood pressure signal in time-domain as the label for supervised learning. As a result, the network can achieve a high continuous blood pressure monitoring accuracy, with the systolic blood pressure root mean square error of 7 mmHg and the diastolic root mean square error of 6 mmHg. These values are within the error range reported in the literature. Note that other methods rely only on mathematical models for the systolic and diastolic values, whereas the proposed method can predict the continuous signal without degrading the measurement performance and relying on a mathematical model.

## 1. Introduction

Blood pressure is the heart’s force to pump blood through the body [1]. This medical parameter is dependent on many physiological and mental factors, such as age, body mass index (BMI), and even stress level [2]. Blood pressure is often measured for two values: systolic and diastolic blood pressure; these numbers are essential parameters for medical analysis. One of the most serious and common conditions that people encounter is having significantly high blood pressure, also known as hypertension [3]. People with hypertension usually have a high chance of encountering potentially fatal conditions such as a stroke or heart attack [4]. In addition, abnormally high blood pressure cannot be cured; nevertheless, it can be managed by a healthy lifestyle change, or by taking medication, and can be prevented by frequently checking the blood pressure [5,6].

In most cases, the systolic and diastolic blood pressure are obtained through a sphygmomanometer [7], which operates based on auscultatory or oscillometric techniques depending on its type [8]. Even though the values can be altered as the cuff position changes, the device’s measured outcomes are still considered highly accurate [9]. However, continuous blood pressure measurement is impractical, and the force applied by the sphygmomanometer’s cuff might be inconvenient for some patients, such as elders. Nowadays, numerous healthcare watches, which can continuously monitor blood pressure, are developed; but the error can vary up to 10 mmHg for systolic and diastolic blood pressure values [10,11,12].

**Figure 1:**
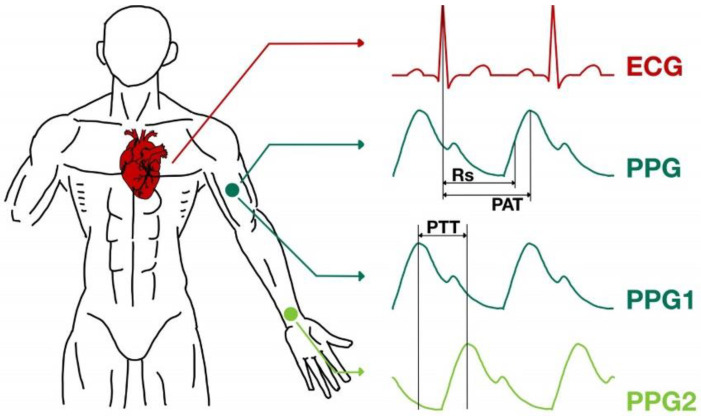
Pulse arrival time (PAT) and pulse transit time (PTT).

Since the cuff method of blood pressure measurement cannot be performed, be recorded and shown in real-time, several alternative methods of predicting blood pressure values have been devised, involving mathematical equations to predict the systolic and diastolic blood pressure value based on a relationship between ECG, PPG, and blood pressure signals [12]. The main parameter for the estimation is either the pulse arrival time (PAT) or pulse transit time (PTT) [13]. The measurements required for PAT and PTT are shown in Figure 1. Nevertheless, other factors such as age, body mass index (BMI), gender, or even posture during the measurement can affect the outcome [13,14,15].

There have been several experiments involving cuff-less blood pressure monitoring [16]. However, most of the research has been based on the pulse transit time (PTT) method [17,18,19,20], and some utilized the pulse arrival time (PAT) method [21,22,23]. In addition, few researchers [24,25] have attempted to predict blood pressure according to photoplethysmography morphology and another physiological partitioning, as shown in Table 1. For example, Wang et al. [26] employed the PAT method with several pulse wave velocity–blood pressure (PWV-BP) models, including the logarithmic, inverse, and inverse square models. Table 1 summarizes the standard deviation of each reviewed method and their optimal measuring ranges.

This research aims to pursue a process that can accurately and effectively provide continuous systolic and diastolic blood pressure measurement through PPG and ECG signals. We propose a pre-signal conditioning method to improve the accuracy of the systolic and diastolic blood pressure measurement by combining the context aggregation network architecture and the preprocessed PPG-ECG signals. It will be shown in the results and discussion later that the proposed method can provide a higher accuracy blood pressure estimation than the other methods reported in the literature for all the ranges of the blood pressures, ranging from 90 mmHg to 180 mmHg, and 60 mmHg to 75 mmHg, for systolic and diastolic blood pressures, respectively. Furthermore, to the best of the authors’ knowledge, the proposed method has never been reported before in the literature.

## 2. Materials and Methods

A context aggregation network (CAN) was employed to demonstrate the accuracy and precision enhancement without adding more parameters to the Equation and reducing noise the ECG and PPG signal. Note that the proposed preprocessing method is not limited to the CAN architecture but is also applicable in other network structures. The CAN network is one of the promising networks for time-series analysis [27] and images analysis [28]. The network’s performance and behavior have been investigated, well understood, and established [29]. Therefore, the CAN architecture was utilized in this study over a newly designed network.

According to the reviewed PAT and PTT, the blood pressure can be approximated on time difference signals. Here, we have utilized the finding in PAT and PTT by randomizing the amplitude of the PPG and EEG signals to ensure that the blood pressure prediction does not rely on the signals’ amplitudes; a CAN with signal Fourier transform and amplitude randomization have been developed and investigated. Here we employed one EEG time-domain signal and one PPG time-domain signal like in the required signals for the PAT method.

### 2.1. ECG and PPG Signal Database

The dataset “Cuff-Less Blood Pressure Estimation Data Set” employed in this study was acquired from the UCI Machine Learning [30]. There were three preprocessed vital signals. The vital signals consist of fingertip photoplethysmography (PPG), electrocardiogram (ECG), and invasive arterial blood pressure (ABP) in the time-domain at a sampling rate of 125 Hz. These signals were monitored and recorded from various hospitals from 2001 to 2008. Furthermore, they were obtained from healthy subjects and patients with pathological conditions, including those with sleep apnea, aging, and movement disorders [30].

### 2.2. Training Dataset and Test Dataset

**Figure 2:**
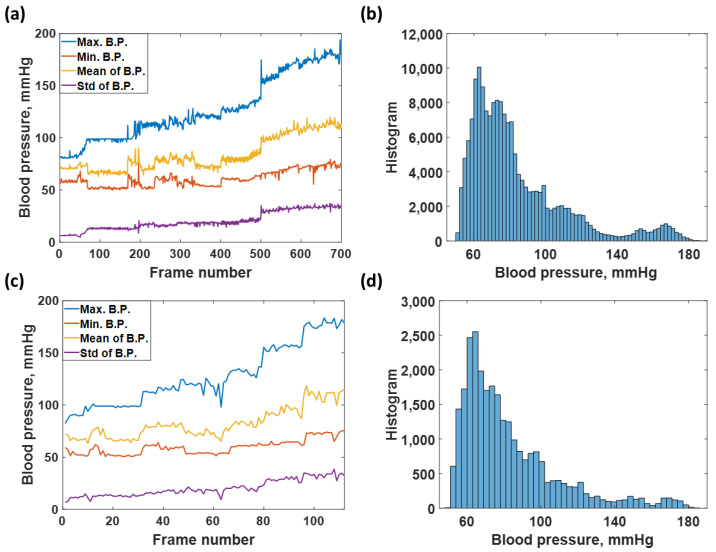
Shows (**a**) average maximum, average minimum, average mean, and standard deviation of each frame number for the training dataset; (**b**) histogram of blood pressure distribution covering in the training dataset; (**c**) average maximum, average minimum, average mean, and standard deviation of each frame number for the test dataset; and (**d**) histogram of blood pressure covering in the test dataset.

The total number of 203,000 data points was extracted from the data source and further separated into a training dataset consisting of 175,000 data points and a test dataset consisting of 28,000 data points. The training dataset was separated into 700 data frames at 250 data points for each frame, representing a 2 s data window for Fourier transformation, explained later. The test dataset was also prepared using the same window size. The training dataset covered all blood pressure levels from 40 to 180, as shown in Figure 2a,b and the test dataset described in Figure 2c,d.

### 2.3. Signal Preconditioning

**Figure 3:**
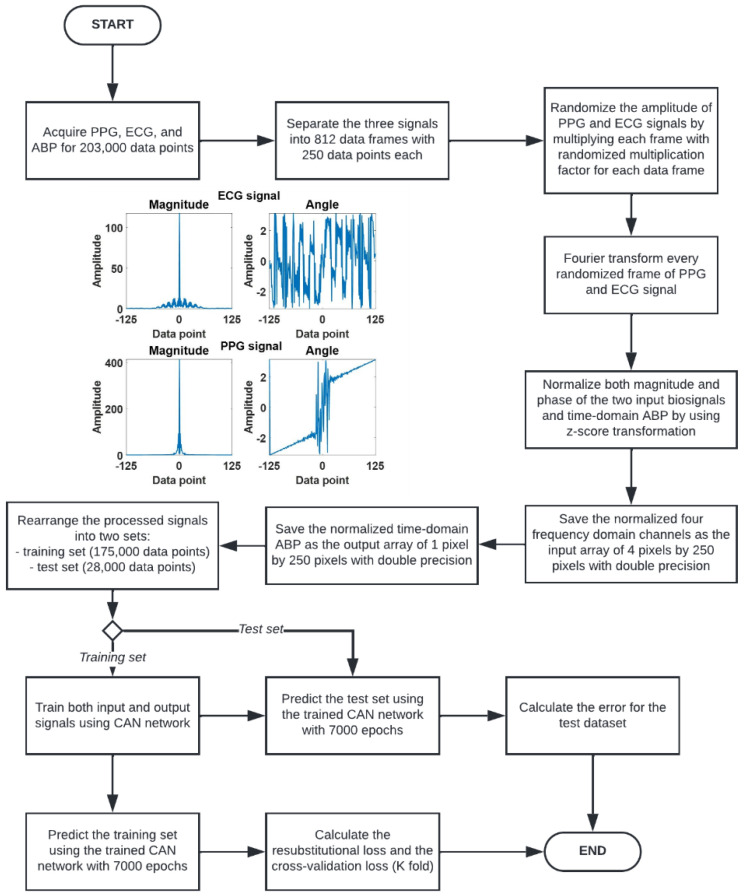
Flowchart describing signal preconditioning process and the network performance analysis.

The signal preconditioning method consists of the following steps to prepare the sequence of data for neural network training, as shown in Figure 3:

1. Acquire the PPG, ECG, and ABP signals for 203,000 data points.
2. Separate the three signals to 203,000 data points into 812 data frames at 250 data points for each data frame. Each frame of the 250 data points represented a 2 s data window.
3. Randomize the amplitude of PPG and ECG signals by multiplying each 2 s window with two randomized multiplication factors for each signal using a uniform random number generator ranging from 0 to 1.
4. Fourier transform every randomized 2 s PPG and ECG signal window. The Fourier transform gives out signals in terms of amplitude and phase, leading to 4 frequency domain channels: the amplitude of ECG, the phase of ECG, the amplitude of PPG, and the phase of PPG, respectively.
5. The four frequency domain channels are then z-score transformed to ensure that the frequency data is appropriate for neural network training. These four channels are then saved as an array of 4 pixels by 250 pixels with double precision.
6. The label of the input arrays is prepared by z-transforming the corresponding ABP signal in the time domain. Note that the input to the CAN was the four channels of frequency-domain data, and the label is the corresponding ABP signal in the time domain.

At the end of step (6), the input consisted of 812 sets of frequency-domain 4 × 250 pixel images and the labels consisted of the corresponding 812 time-domain arterial blood pressures of 1 × 250 pixels. The 812 sets were then separated into 3 datasets using random selection, which comprised 665 training data, 35 validation data and 112 test data.

### 2.4. Context Aggregation Neural Network (CAN) Training

The Context Aggregation Neural Network (CAN) is one of the Convolutional Neural Networks (CNN), a deep learning algorithm that works based on a mathematical operation called convolution. The CAN network consists of only one stage that performs both classification and forecasting processes concurrently. Generally, this network is used for image processing, such as noise reduction [31], and learnable weights and biases can be defined to differentiate the input image. 

**Figure 4:**
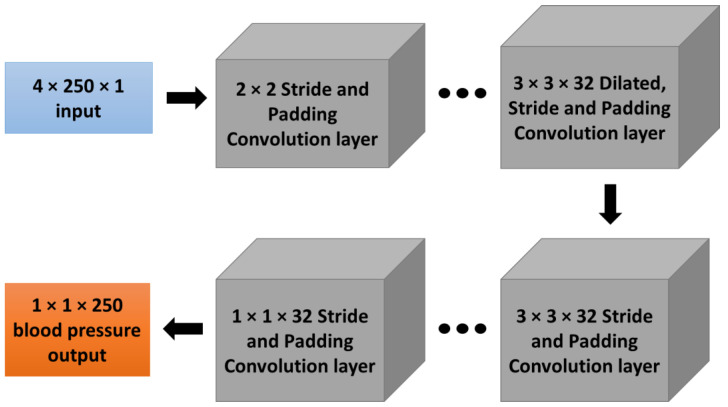
Context Aggregation Network (CAN) architecture with 4 × 250 data inputs for both magnitude and phase of ECG and PPG (4 × 250 pixels) in the frequency domain and 250 data outputs of blood pressure (1 × 250 pixels) in the time domain.

However, even though it can provide excellent image classification performance, it requires much data and time to obtain high output accuracy [32]. Therefore, this study applied the CAN with the network architecture in Figure 4 to train the described training dataset and test the regression accuracy using the test set.

The frequency-domain for both magnitude and phase of ECG and PPG signals are converted to a 4 × 250 pixels image and used as training input, while the frequency-domain blood pressure signal is reconstructed as a 1 × 250 pixel image and used as a training label. According to the pulse arrival time (PAT), the calculated systolic and diastolic blood pressure does not rely on ECG and PPG data amplitude. Therefore, the prepared input signals are multiplied with a random number for each of the 250 data points. Numerous sets of input and output images are used to train the context aggregation network (CAN); complete details of the implemented networks are shown in Table 2.

The CAN network can assign learnable weights and biases by the adaptive normalization layer, while the Batch normalizer is used to overfit the neural network by using the mean and standard deviation of data. In this case, the implemented network consists of ten layers. For the first to eighth layer, the layer consists of padding functions and dilation filters with sizes increasing exponentially to 128 at the eighth layer; the output size of each layer will be identical, which is 4 × 250 × 32 pixels. The ninth layer consists of one padding function and stride, and the output from this layer is the same as the upper layer and will be applied to the regression layer. The ninth layer is the last convolutional layer used to transform the output size to be 4 × 250 × 3 pixels for entering the regression layer. Finally, the tenth layer is a regression layer which consists of a 3 × 3 filter that transforms the output size to be 1 × 250 pixels, which will be the predicted blood pressure data for 2 s (sampling frequency of 125 Hz). This context aggregation neural network (CAN) is trained with a 0.0001 learning rate for 7000 epochs of training iteration using MATLAB2021c with graphic processing unit (GPU) NVIDIA GeForce GTX 1070, using the training process described in Figure 3.

The hyperparameters for the training are as summarized in Table 3. The network was designed to cope with the overfitting issue by adding multiple stages of batch normalization layers, as shown in Table 2. It will be shown in the next section that the slight overfitting of 4.5 mmHg is well within the RMSE of root mean square errors (RMSE). Therefore, further network architecture modification is unnecessary since the predicted response is already within the error limit. 

There are two types of RMSEs investigated to evaluate the network performance in this study:

1. Resubstitution RMSE is computed by evaluating the root mean square error between the training labels and outputs predicted with the training data using the network trained using the labels and the training dataset [33,34,35]. In other words, how accurately the network can predict the labels of the training dataset.
2. Cross-validation RMSE or K-fold cross-validation; the network performance is evaluated using validation K-fold of 5 by separating the training and validation dataset into five sub-datasets. Note that the members of each sub-datasets were chosen at random and then trained five separate networks using each sub-dataset. The cross-validation RMSE was then computed as the average RMSE error of the five networks.

The main difference between the two RMSEs is that the resubstitution RMSE provides an overall performance, whereas the cross-validation RMSE can provide an insight into how the noises and discrepancies in the dataset affect the trained network performance. Here, the two RMSEs will be quantified and discussed.

## 3. Results

### 3.1. Network Training

**Figure 5:**
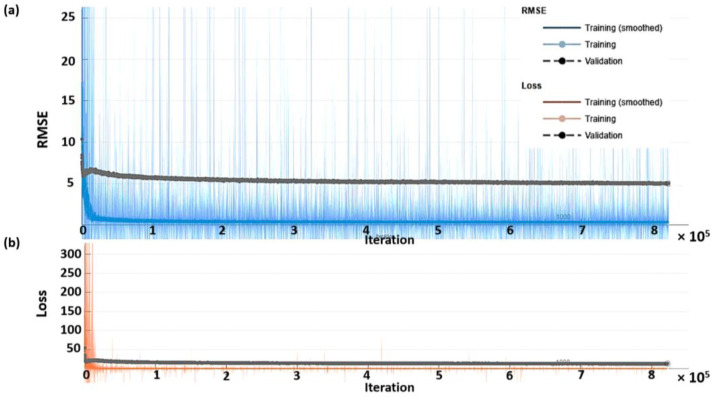
Shows the network training and validation for different iterations ranging from 0 to 82,500 epochs (**a**) RMSE in mmHg, and (**b**) loss; training RMSE is the blue curve, training loss is the red curve, and validation curves are shown in black.

Figure 5 shows the RMSE for each training epoch and the loss in mmHg. The training dataset was further separated into 95% and 5% for training and validation. The training response is displayed in the blue curve for the RMSE, and the validation RMSE is shown in the black curve, as shown in Figure 5a. The training was carried to 7000 epochs taking around 26 h under the single GPU environment, as described in detail in the materials and methods section. Figure 5b shows the loss from the training for the same range of epochs. After the training, the network was slightly overfitting by 4.5 mmHg when comparing the training curve to the validation curve, and the training has converged to its stable response. The network was designed to cope with the overfitting issue by adding multiple stages of batch normalization layers, as shown in Table 2. It will be shown in the next section that the slight overfitting of 4.5 mmHg is well within the RMSE of resubstitution error and the cross-validation error. Therefore, further network architecture modification was unnecessary since the predicted response was already within the error limit.

The loss reported in Figure 5b was computed using the function using half mean square error expressed in Equation (1).

(1) $$loss=\frac{1}{2}\overset{L}{\underset{p=1}{\sum }}{\left(t_{p}-y_{p}\right)}^{2}$$

where *loss* is the half mean square loss function, and *L* is the output sequence length corresponding to 250 data points. The terms *t_p_* and *y_p_* are the training labels and the predicted responses at the *p*th pixel of the output sequence. 

### 3.2. Blood Pressure Prediction of the Trained Sequences

**Figure 6:**
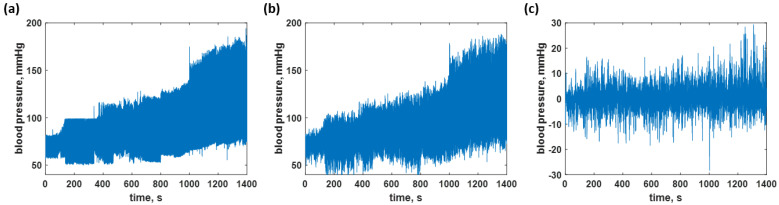
Shows the blood pressure levels in (**a**) the training dataset; (**b**) resubstitution prediction using the trained network; and (**c**) resubstitution loss calculated using the data in Figure 6a,b.

**Figure 7:**
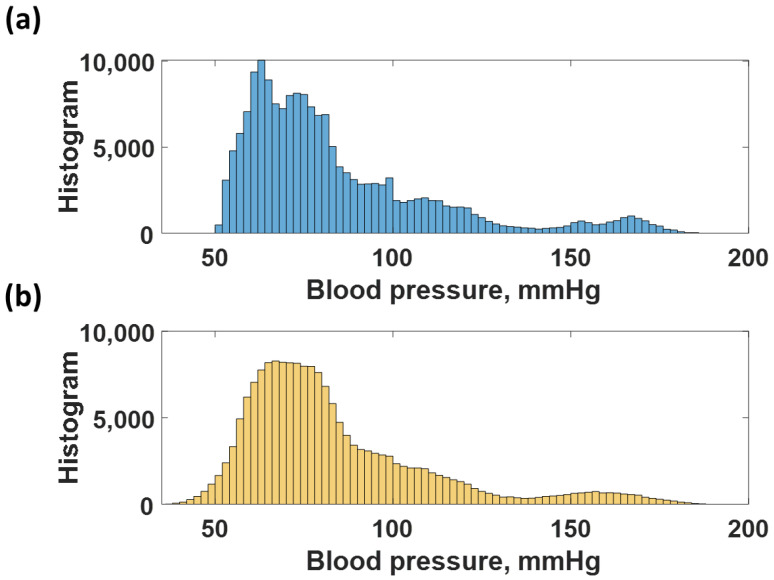
Shows histogram of (**a**) training dataset, and (**b**) the resubstitution responses predicted using the training dataset and the trained CAN network.

The CAN network was trained using the data shown in Figure 6a, as explained in the earlier section. The trained network was then employed to predict the time-domain data of all the training data to calculate the resubstitution loss. The predicted responses from the network are shown in Figure 6b; meanwhile, Figure 6c shows the resubstitution error calculated by subtracting the time-domain B.P. signals in Figure 6a,b. The corresponding resubstitution RMSE was 4.27 mmHg, similar to the RMSE of the validation. Although the overall shape of the resubstitution B.P. signal agreed well with the training dataset, the fluctuation of the time domain data had a higher range, as shown in the histogram in Figure 7b compared to the distribution of the training dataset shown in Figure 7a.

**Figure 8:**
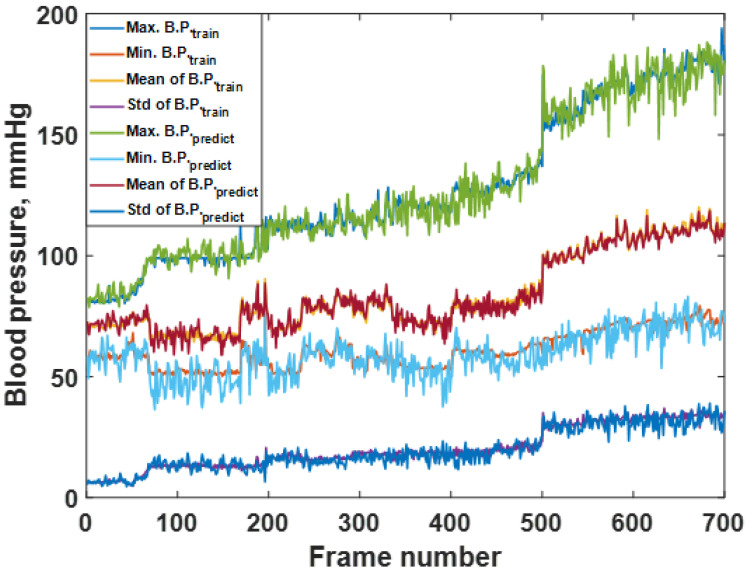
Shows the average max, min, mean, and standard deviation of each frame number for the training dataset compared to the predicted responses.

The predicted responses in Figure 6a,b can be further analyzed by calculating the average maximum B.P., the average minimum B.P, the average mean B.P., and the standard deviation, as discussed in the materials and methods section. Figure 8 shows the average maximum B.P., the average minimum B.P, the average mean B.P., and the standard deviation calculated for the 700 frames of the training dataset and predicted response. The frames’ trends between the training dataset and the predicted response agreed well. The RMSE values between the training and the resubstitution response were 4.9590, 5.0880, 1.9776, and 2.4064 mmHg for the average maximum B.P., the average minimum B.P, the average mean B.P., and the standard deviation, respectively. The RMSE values for the systolic and the diastolic blood pressures were around 5 mmHg, with a slight error for the overall mean value within 2 mmHg. In the case of standard deviation RMSE, 2.4064 mmHg indicates that the predicted B.P. levels using the trained CAN network did not deviate from the training data much. For the K-fold cross-validation, the RMSE values were 6.8750, 7.0772, 2.0125, and 2.6211 mmHg for the average maximum B.P., the average minimum B.P, the average mean B.P., and the standard deviation, respectively. It can be seen that the discrepancies between the two types of RMSE are well within 2 mmHg, indicating the proposed preprocessing method has enabled the trained network to be more robust to temporal amplitude fluctuations. It will be discussed later that if the network is trained without the preprocessing method, its performance will be worse and heavily affected by amplitude fluctuations.

**Figure 9:**
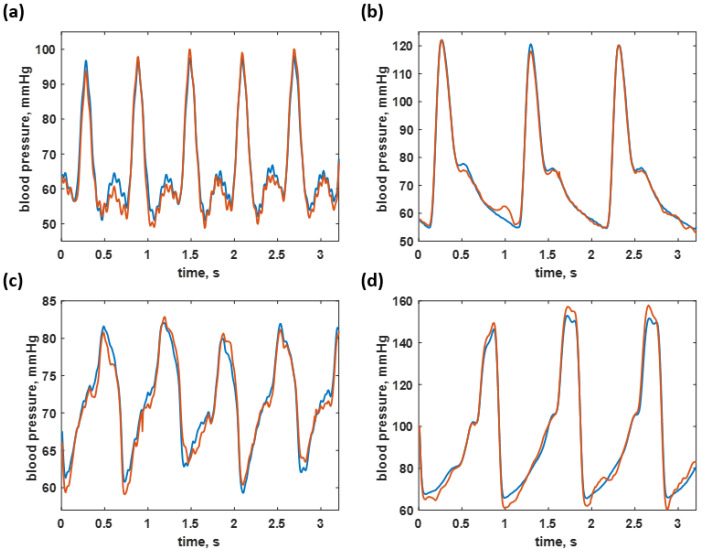
Shows the blood pressure predicted by the trained neural network (red curves) compared to the actual blood pressure signal (blue curves) across four ranges: (**a**) 108/55 mmHg (healthy); (**b**) 125/60 mmHg (healthy); (**c**) 79/58 mmHg (hypotension); and (**d**) 150/67 mmHg (hypertension).

Figure 9 shows some examples of 250 datapoint frames covering the blood pressure of healthy individuals of 108/55 mmHg and 125/60 mmHg, as shown in Figure 9a,b, hypotension case at the B.P. level of 79/58 mmHg, shown in Figure 9c, and a hypertension B.P. level of 150/67 mmHg, shown in Figure 9d. It can be seen that the predicted values agree well with the training data. The predicted signals were highly accurate for the healthy and hypotensive blood pressure ranges. However, the hypertensive blood pressure estimation has resulted in a moderate resubstitution loss. The trained network was then applied to predict the untrained test dataset to see how well the network could perform in the next section.

### 3.3. Blood Pressure Prediction of the Test Dataset

**Figure 10:**
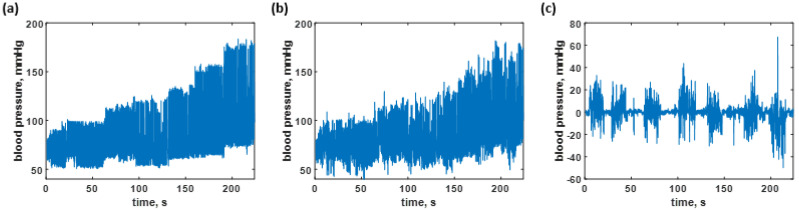
Shows the blood pressure levels in (**a**) the test dataset; (**b**) B.P. prediction using the trained network; and (**c**) test data error calculated using the data in Figure 7a,b.

**Figure 11:**
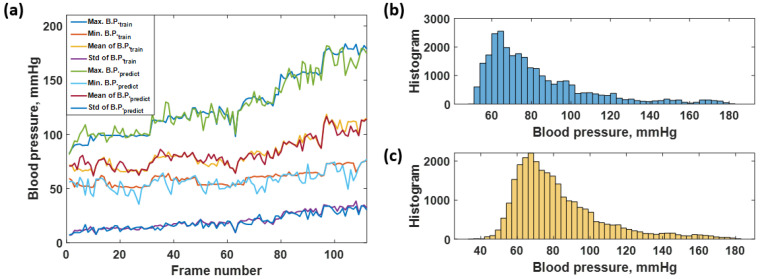
Shows the (**a**) average max, min, mean, and standard deviation of each frame number for the test dataset and predicted responses; (**b**) histogram of blood pressure level covering in the actual testing blood pressure data; and (**c**) histogram of blood pressure level covering in the predicted blood pressure data.

The blood pressure levels in the test dataset consisted of 28,000 data points which contain the same level of blood pressure as the training sequence from 40 to 180 mmHg, as shown in Figure 10a and depicted in the histogram shown in Figure 11b. Therefore, the predicted sequence is shown in Figure 10b and depicted as the histogram in Figure 11c, while the regression error is shown in Figure 10c. Figure 10c corresponds to the overall RMSE of 7.73 mmHg. The predicted response can also be further analyzed by analyzing individual frames of the 250 datapoint window to determine what was causing the discrepancies. Figure 11a shows the average maximum B.P., the average minimum B.P, the average mean B.P. and the standard deviation for the label of the test dataset and the predicted responses using the trained network. The healthy blood pressure signal had the most accurate prediction for both systolic and diastolic values, while the hypotension and extreme hypertension cases signal yielded a high degree of error, as shown in Figure 11a. There was no trend difference between the label and predicted blood pressure, but the predicted B.P. fluctuated more than the label. The RMSE values were then calculated comparing the label to the predicted values and yielded 7.1455, 6.0862, 4.2381, and 2.3218 mmHg for systolic, diastolic, mean, and standard deviation of blood pressure, respectively. Therefore, it shows that the average maximum or systolic blood pressure gives the highest error, around 7 mmHg, compared to the diastolic and mean blood pressure data. The small standard deviation RMSE, 2.3218 mmHg, indicated that the overall performance was acceptable, and the predicted blood pressure did not deviate much. Comparing the four types of RMSE calculated from the training blood pressure dataset and the testing blood pressure dataset, the systolic, diastolic, and the mean RMSE of the test dataset is higher than the training dataset around 1–3 mmHg. The standard deviation RMSE of both cases was similar.

For the K-fold cross-validation, the RMSE values were 9.1287, 8.0812, 4.3522, and 2.4317 mmHg for the average maximum B.P., the average minimum B.P, the average mean B.P., and the standard deviation, respectively. The K-fold RMSEs were slightly more than the RMSEs reported for the test dataset; they were well within 2 mmHg, as in the training dataset. The proposed preprocessing method can provide a robust network to signal amplitude noises. Section 3.3 will demonstrate that networks trained without the preprocessing method have a worse performance for the test dataset.

**Figure 12:**
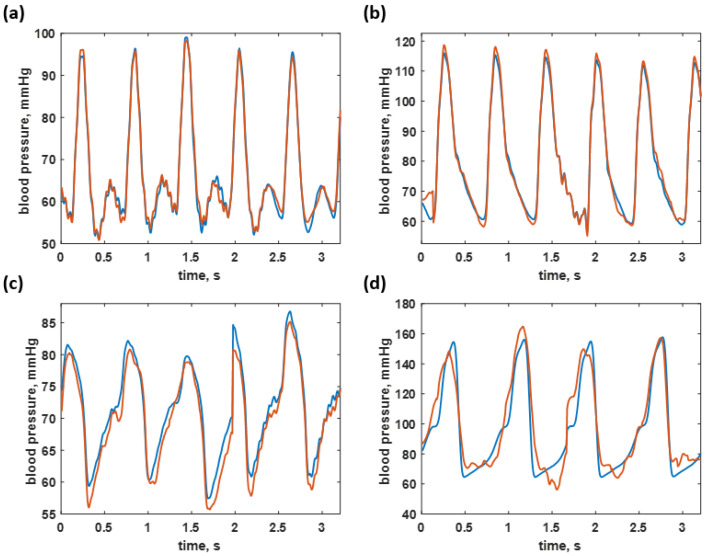
Shows the blood pressure predicted by the trained neural network (red curves) compared to the untrained actual blood pressure signal (blue curves) across four ranges: (**a**) 95/51 mmHg (healthy); (**b**) 110/62 mmHg (healthy); (**c**) 78/53 mmHg (hypotension); and (**d**) 179/70 mmHg (hypertension).

Figure 12 shows some examples of 250 datapoint frames covering the blood pressure of healthy individuals of 95/51 mmHg and 110/62 mmHg, as shown in Figure 12a,b, hypotension case at the B.P. level of 78/53 mmHg, shown in Figure 12c, and a hypertension B.P. level of 179/70 mmHg, shown in Figure 12d. The test dataset predictions were only accurate in a healthy range, while the hypotension and hypertension cases had slightly higher discrepancies, as shown in Figure 12a–d for the healthy cases and Figure 12c–d for the hypotension and hypertension cases, respectively. Although the maximum discrepancy was around 7 mmHg, the CAN network can still provide an overall correct response; this can open up another approach to continuously monitor the time-domain blood pressure signal, with the response slightly worse than home-use digital blood pressure devices in the market. Shahbabu et al. [36] reported that the absolute error between the digital sphygmomanometer and the mercury-based sphygmomanometer was 5 mmHg.

### 3.4. Performance Comparison to Networks Trained without the Proposed Preprocessing Method

This section demonstrates that the proposed preprocessing method can enhance the network’s robustness to the amplitude noise signal. Here, a long short-term memory (LSTM) network with the network architecture is summarized in Table 4. The LSTM network is well-known for time series analysis. The LSTM employed in this study consisted of 2 input channels for the time-domain ECG and PPG signals 2 × 175,000 data points and 1 response (output) channel 1 × 175,000 data points for the time-domain arterial blood pressure. The network was trained using the same dataset as the CAN network with a 0.005 learning rate for 7000 epochs of training iteration using MATLAB2021c and the GPU NVIDIA GeForce GTX 1070. Note that here the training required slightly different hyperparameters than the CAN training due to the memory limitation of the GPU, as shown in Table 5.

**Figure 13:**
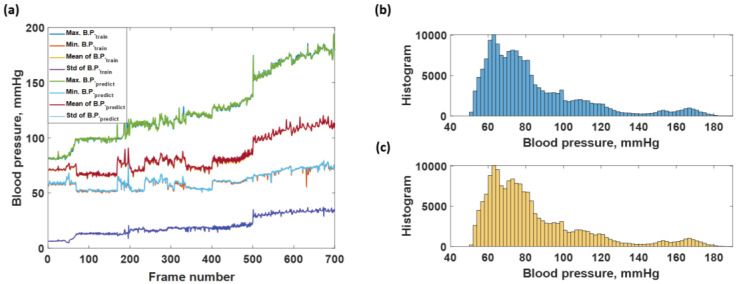
Shows the (**a**) average max, min, mean, and standard deviation of each frame number training dataset and predicted responses; (**b**) histogram of blood pressure level covering in the actual training blood pressure data; and (**c**) histogram of blood pressure level covering in the predicted blood pressure data by using LSTM network.

The same training dataset trained the LSTM network as the CAN network explained in the previous section. Figure 13 shows that the predicted blood pressure of the trained data is highly accurate compared to the labels. The RMSEs calculated by comparing the labels to the predicted blood pressures, as depicted in Figure 13a, were 0.6804, 0.8556, 0.3992, and 0.1348 mmHg for systolic, diastolic, mean, and standard deviation, respectively. In addition, the histogram of the labels shown in Figure 13b and the predicted blood pressures shown in Figure 13c agree well with each other, indicating the LSTM network had a decent resubstitution prediction performance.

**Figure 14:**
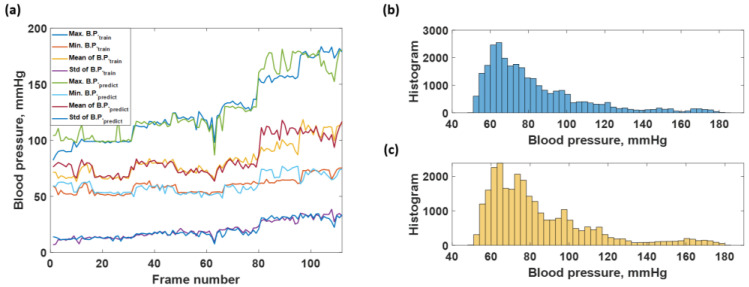
Shows the (**a**) average max, min, mean, and standard deviation of each frame number for test dataset and predicted responses; (**b**) histogram of blood pressure level covering in the actual testing blood pressure data; and (**c**) histogram of blood pressure level covering in the predicted blood pressure data by using LSTM network.

The test dataset for LSTM consisted of 28,000 data points used in the CAN network prediction, as explained in Section 3.2. The test dataset predictions are shown in Figure 14a. It contained the same blood pressure level as the training sequence ranging from 40 to 180 mmHg. In contrast to the resubstitution performance, the predicted blood pressure for the test dataset had a significantly worse regression accuracy with the RMSEs of 9.5528, 5.3774, 7.2500, and 2.5795 mmHg for maximum, minimum, mean, and standard deviation, indicating that the LSTM suffered from noise in the time-domain amplitude signals. Figure 14b,c shows histograms of the test dataset and the predicted responses, respectively. The histogram of the predicted responses noticeably differed from the test dataset.

Table 6 summarizes the RMSE results comparing the CAN network with the proposed preprocessing method to the LSTM network without the preprocessing method. For the resubstitution performance, the LSTM prediction was predominantly more accurate than the CAN network for all RMSE calculations. For the LSTM resubstitution performance, all RMSE values were less than 1 mmHg.

Nevertheless, the test dataset prediction performance of the CAN network was more precise than the LSTM prediction. The performance of the LSTM drastically deteriorated to the RMSE levels higher than the CAN responses, as shown in Table 6. The CAN with the proposed preprocessing method can enhance the network robustness to noise and amplitude fluctuations. The following section will show that the CAN network can predict a continuous blood pressure signal from ECG and PPG data from other data sources and discuss the CAN network’s performance, advantages and limitations compared to the conventional methods, including PAT and PPT.

### 3.5. The CAN Network Prediction Compared to Other Methods

Here, another dataset recording EEG, PPG, systolic blood pressure, and diastolic blood pressure reported by Wang et al. [26] was adopted to demonstrate the capability and adaptability of other proposed method datasets compared to conventional methods including conventional methods the PAT and PTT. Wang et al. [26] reported that accurately estimating the systolic blood pressure and diastolic blood pressure depended on the mathematical model and preprocessing methods. The standard deviation for the systolic blood pressure reported by Wang et al. [26] was 7.736 mmHg to 8.793 mmHg, and 3.448 mmHg to 3.622 mmHg, for the systolic blood pressure and diastolic blood pressure, respectively. By analyzing the example dataset in Wang et al. [26], the CAN network can predict the systolic blood pressure and diastolic blood pressure to the RMSE of 7.0252 mmHg and 5.217 mmHg for systolic and diastolic blood pressures.

By comparing the mean and standard deviation error to the reviewed methods in Table 1 and Wang et al. [26], the proposed signal preconditioning method can perform within the range of the error reported by the others. For example, the highest systolic error was 7.1455 mmHg, lower than the systolic error obtained by any PWV-BP models.

It is crucial to point out the advantage of the proposed method. Firstly, it is a deep learning approach, which requires no model or known equations. Secondly, our proposed method can predict the real-time blood pressure signal compared to the other methods that only calculate the systolic and diastolic blood pressure values. The proposed method can open up another approach for cuff-less blood pressure monitoring in real-time for a smartwatch. However, the developed CAN network for blood pressure monitoring still has limitations, especially the higher RMSE in the diastolic blood pressure estimation than the systolic blood pressure. This is due to the limited access to the data for training. The proposed method is not a good candidate for real-time monitoring using a smartwatch, since it requires continuous signal processing and a GPU for the deep learning-based software. 

The limitations, however, do not obscure the main objective and the key benefit of the proposed preprocessing method that the proposed method can train the network to be more robust to noise in amplitude signals. The feature is achieved by training the network using randomized amplitude and frequency-domain signals instead of the time-domain signal. The proposed method can be applied in applications that suffer from signal amplitude noise, such as optical biosensors [28,37], biomechanics [38], including human body movement and tracking, and energy transformation in engineering fields [39,40].

## 4. Conclusions

The studies have shown the mathematical relationship between ECG, PPG, and the blood pressure signals, which can be utilized as an alternative way of a cuff-less measurement of the systolic and diastolic blood pressure. Therefore, we proposed a signal preconditioning method to prepare a dataset for deep learning training. The proposed method consisted of the following steps: (1) acquiring the time-domain ECG, PPG, and ABP signals at 125 Hz; (2) grouping the time-domain ECG and PPG signals into data blocks of 250 sampling points corresponding to 2 s intervals; (3) multiplying the 2 s window frames with two randomized multiplication factors with their values ranging from 0 to 1 to ensure that the data does not contain amplitude information, since the amplitude of ECG and PPG can fluctuate widely due to several possible reasons, for example, electrode attachment, the detector battery and source; (4) Fourier transforming the PPG and EEG frames in (3), obtaining four input signals: the amplitude and the phase of PPG, and the amplitude and the phase of EEG; (5) then, normalizing the four input signals using z-transform; and finally, (6) training the CAN network using the four frequency domain signals and their corresponding arterial blood pressure in the time-domain as the label. The network was trained using 700 frames (175,000 data points) and tested using 112 frames of the untrained dataset covering blood pressure from 80 mmHg to 180 mmHg. The trained network can provide an accurate prediction compared to the test label with the RMSE values of 7.1455, 6.0862, 4.2381, and 2.3218 mmHg for systolic, diastolic, mean, and standard deviation of blood pressure, respectively. The K-fold cross-validation using K-fold of 5 also shows a similar trend of the RMSEs, indicating that the proposed method enables the network to learn from the frequency distributions and is more to temporal noises in the time-domain signals. The advantages of the proposed method include (1) it is a deep learning approach, and of course, did not require a known mathematical model. (2) The performance and accuracy are similar to the other cuff-less blood pressure monitor reported in the literature. Moreover, (3) the proposed method can provide a continuous time-domain B.P. signal, potentially providing a convenient means for continuous cuff-less blood pressure monitoring. The proposed method has also been tested for its prediction performance using the dataset from the other source and found that the trained network can be employed to predict the continuous blood pressure of the outside dataset at the same range of RMSEs. There are still some limitations, including the lower prediction accuracy for the diastolic blood pressure than the systolic blood pressure. The proposed continuous blood pressure monitoring is not applicable for a smartwatch due to the GPU requirement for software and the continuous signal processing, which can quickly drain the battery. The proposed preprocessing method can train deep learning using frequency-domain signals for more robust noise amplitude fluctuations and enhance performance in applications that suffer from temporal amplitude noise.

## Figures and Tables

**Table 1:** Accuracy of previous blood pressure estimations based on PTT, PAT, and other methods.

	Systolic Blood Pressure Errors (Mean ± Standard Deviation)	Diastolic Blood Pressure Errors (Mean ± Standard Deviation)	Operating Range Systolic Blood Pressure	Operating Range Diastolic Blood Pressure
**PTT-based Methods**				
Two-step algorithm developed by machine learning [17]	0.07 ± 7.1 mmHg	−0.08 ± 6.0 mmHg	Reduced accuracy for hypotension	Reduced accuracy for hypotension
B.P. estimation based on PTT and photoplethysmography intensity ratio (PIR) [18]	−0.37 ± 5.21 mmHg	−0.08 ± 4.06 mmHg	Reduced accuracy for hypertension	Reduced accuracy for hypertension
B.P. estimation based on PTT and intensity ratio of the first derivative wave of PPG (1st-dPIR) [19]	2.88 ± 7.75 mmHg	2.80 ± 4.38 mmHg		
Proceeding PTT-based method on the repeatability test [20]	0.0 ± 5.3 mmHg	0.0 ± 2.9 mmHg	80–150 mmHg	60–120 mmHg
Proceeding PTT-based method using regression coefficients [20]	1.4 ± 10.2 mmHg	2.1 ± 7.3 mmHg	80–150 mmHg	60–120 mmHg
**PAT-based Methods**				
Estimating beat-by-beat blood pressure using Chen’s method [21]	−0.5 ± 5.3 mmHg	4.1 ± 3.4 mmHg		
Standard pulse arrival time based method calculations [22]	0 ± 3 mmHg	0 ± 3 mmHg		
Using a linear correlation of systolic blood pressure and a non-linear correlation of diastolic blood pressure and PAT [23]	0.2 ± 5.8 mmHg	0.4 ± 5.7 mmHg		
Model-driven method: Logarithmic [26]	−0.512 ± 8.793 mmHg	−0.148 ± 3.622 mmHg		
Model-driven method: Inverse [26]	−0.008 ± 8.203 mmHg	−0.078 ± 3.448 mmHg		
Model-driven method: Inverse Square [26]	−0.358 ± 8.084 mmHg	−0.066 ± 3.574 mmHg		
**Other Methods**				
Estimating blood pressure based on pulse morphology of PPG [24]	0.043 ± 5.001 mmHg	0.011 ± 3.689 mmHg		
Blood pressure prediction based on demographic and physiological partitioning [25]	Mean absolute error = 6.9 mmHg	Mean absolute error = 5 mmHg	80–220 mmHg	45–120 mmHg

**Table 2:** Details of the implemented context aggregation network.

Layer	Activations	Learnable Variable	Descriptions
Image input	4 × 250 × 1	–	4 × 500 × 1 images
Convolutional	4 × 250 × 32	Weights 2 × 2 × 1 × 32, Bias 1 × 1 × 32	1 padding
Batch normalization		Offset 1 × 1 × 32, Scale 1 × 1 × 32	–
Adaptive normalization		–	–
Leaky ReLU		–	Scale 0.2
Convolutional		Weights 3 × 3 × 32 × 32, Bias 1 × 1 × 32	2 padding, 1 Stride, 2 dilation
Batch normalization		Offset 1 × 1 × 32, Scale 1 × 1 × 32	–
Adaptive normalization		–	–
Leaky ReLU		–	Scale 0.2
Convolutional		Weights 3 × 3 × 32 × 32, Bias 1 × 1 × 32	4 padding, 1 Stride, 4 dilation
Batch normalization		Offset 1 × 1 × 32, Scale 1 × 1 × 32	–
Adaptive normalization		–	–
Leaky ReLU		–	Scale 0.2
Convolutional		Weights 3 × 3 × 32 × 32, Bias 1 × 1 × 32	8 padding, 1 Stride, 8 dilation
Batch normalization		Offset 1 × 1 × 32, Scale 1 × 1 × 32	–
Adaptive normalization		–	–
Leaky ReLU		–	Scale 0.2
Convolutional		Weights 3 × 3 × 32 × 32, Bias 1 × 1 × 32	16 padding, 1 Stride, 16 dilation
Batch normalization		Offset 1 × 1 × 32, Scale 1 × 1 × 32	–
Adaptive normalization	4 × 250 × 32	–	–
Leaky ReLU		–	Scale 0.2
Convolutional		Weights 3 × 3 × 32 × 32, Bias 1 × 1 × 32	32 padding, 1 Stride, 32 dilation
Batch normalization		Offset 1 × 1 × 32, Scale 1 × 1 × 32	–
Adaptive normalization		–	–
Leaky ReLU		–	Scale 0.2
Convolutional		Weights 3 × 3 × 32 × 32, Bias 1 × 1 × 32	64 padding, 1 Stride, 64 dilation
Batch normalization		Offset 1 × 1 × 32, Scale 1 × 1 × 32	–
Adaptive normalization		–	–
Leaky ReLU		–	Scale 0.2
Convolutional		Weights 3 × 3 × 32 × 32, Bias 1 × 1 × 32	128 padding, 1 Stride, 128 dilation
Batch normalization		Offset 1 × 1 × 32, Scale 1 × 1 × 32	–
Adaptive normalization		–	–
Leaky ReLU		–	Scale 0.2
Convolutional		Weights 3 × 3 × 32 × 32, Bias 1 × 1 × 32	1 padding, 1 Stride
Batch normalization		Offset 1 × 1 × 32, Scale 1 × 1 × 32	–
Adaptive normalization		–	–
Leaky ReLU	4 × 250 × 32	–	Scale 0.2
Convolutional	4 × 250 × 3	Weights 3 × 3 × 32 × 32, Bias 1 × 1 × 32	0 padding, 1 Stride
Regression output	1 × 1 × 250	–	Mean square error

**Table 3:** Details of the implemented context aggregation network.

Hyperparameter	Parameter Value
Initial Learn Rate	$1\times 10^{-4}$
Gradient Decay Factor	0.9000
Squared Gradient Decay Factor	0.9990
Epsilon (ε)	$1\times 10^{-8}$
Learn Rate Schedule	piecewise
Learn Rate Drop Factor	0.0100
Learn Rate Drop Period	125,000
L2 Regularization	$1\times 10^{-4}$
Gradient Threshold Method	L2 norm
Gradient Threshold	1
Maximum Epochs	7000
Mini Batch Size	1
Input and Label Shuffle	every epoch

**Table 4:** Details of the implemented Long Short-Term Memory network.

Layer	Activations	Learnable Variable	Descriptions
Sequence input	2	–	Sequence input with 2 dimensions
LSTM	400	InputWeights 1600 × 2, RecurrentWeights 1600 × 400, Bias 1600 × 1	LSTM with 400 hidden units
Fully Connected	1	Weights 1 × 400, Bias 1 × 1	1 fully connected layer
Regression Output	1	–	Mean-squared-error with response

**Table 5:** Details of the hyperparameters used in the Long Short-Term Memory network.

Hyperparameter	Parameter Value
Initial Learn Rate	$5\times 10^{-3}$
Gradient Decay Factor	0.9000
Squared Gradient Decay Factor	0.9990
Epsilon (ε)	$1\times 10^{-8}$
Learn Rate Schedule	piecewise
Learn Rate Drop Factor	0.0100
Learn Rate Drop Period	125,000
L2 Regularization	$1\times 10^{-4}$
Gradient Threshold Method	L2 norm
Gradient Threshold	1
Maximum Epochs	7000
Mini Batch Size	2
Input and Label Shuffle	once

**Table 6:** The performance comparison between the CAN with the proposed preprocessing method and the LSTM without the preprocessing method.

**RMSEs for the Resubstitution Performance**		
**RMSE**	**CAN with the Preprocessing**	**LSTM without the Preprocessing**
The average maximum B.P.	4.9590 mmHg	0.6804 mmHg
The average minimum B.P	5.0880 mmHg	0.8556 mmHg
The average mean B.P.	1.9776 mmHg	0.3992 mmHg
The standard deviation	2.4064 mmHg	0.1348 mmHg
**RMSEs for the Test Dataset Responses**		
**RMSE**	**CAN with the Preprocessing**	**LSTM without the Preprocessing**
The average maximum B.P.	7.1455 mmHg	9.5528 mmHg
The average minimum B.P	6.0862 mmHg	7.3774 mmHg
The average mean B.P.	4.2381 mmHg	7.2500 mmHg
The standard deviation	2.3218 mmHg	2.5795 mmHg

## Data Availability

The paper analyzed two open-source datasets downloaded from https://archive.ics.uci.edu/ml/datasets/Cuff-Less+Blood+Pressure+Estimation (accessed on 10 December 2021) and https://drive.google.com/file/d/1c8_4sXGxTVxROt4tiTd-lYC1hPvCEaHj/view (accessed on 10 December 2021).
